# Assessment of the influence of ABO blood groups on oral cholera vaccine immunogenicity in a cholera endemic area in Zambia

**DOI:** 10.1186/s12889-023-15051-w

**Published:** 2023-01-23

**Authors:** Caroline C. Chisenga, Samuel Bosomprah, Obvious N. Chilyabanyama, Peter Alabi, Michelo Simuyandi, John Mwaba, Harriet Ng’ombe, Natasha M. Laban, Charlie C. Luchen, Roma Chilengi

**Affiliations:** 1 Centre for Infectious Disease Research in Zambia, Lusaka, Zambia; 2 Department of Biostatistics, School of Public Health, University of Ghana, Accra, Ghana; 3 Department of Biomedical Sciences, School of Health Sciences, University of Zambia, Lusaka, Zambia; 4 School of Medicine, University of Lusaka, Lusaka, Zambia

**Keywords:** Cholera, Histo-blood group antigens, Secretory status, Vaccine response, Zambia

## Abstract

**Background:**

Histo-blood group antigens (HBGAs) which include the ABO and Lewis antigen systems have been known for determining predisposition to infections. For instance, blood group O individuals have a higher risk of severe illness due to *V. cholerae* compared to those with non-blood group O antigens. We set out to determine the influence that these HBGAs have on oral cholera vaccine immunogenicity and seroconversion in individuals residing within a cholera endemic area in Zambia.

**Methodology:**

We conducted a longitudinal study nested under a clinical trial in which samples from a cohort of 223 adults who were vaccinated with two doses of Shanchol™ and followed up over 4 years were used. We measured serum vibriocidal geometric mean titers (GMTs) at Baseline, Day 28, Months 6, 12, 24, 30, 36 and 48 in response to the vaccine. Saliva obtained at 1 year post vaccination was tested for HBGA phenotypes and secretor status using an enzyme-linked immunosorbent assay (ELISA).

**Results:**

Of the 133/223 participants included in the final analysis, the majority were above 34 years old (58%) and of these, 90% were males. Seroconversion rates to *V. cholerae* O1 Inaba with non-O (23%) and O (30%) blood types were comparable. The same pattern was observed against O1 Ogawa serotype between non-O (25%) and O (35%). This trend continued over the four-year follow-up period.

Similarly, no significant differences were observed in seroconversion rates between the non-secretors (26%) and secretors (36%) against *V. cholerae* O1 Inaba. The same was observed for O1 Ogawa in non-secretors (22%) and the secretors (36%).

**Conclusion:**

Our results do not support the idea that ABO blood grouping influence vaccine uptake and responses against cholera.

## Introduction

Zambia is among countries in sub-Saharan Africa contributing to 2.9 million cases and 95,000 deaths resulting from cholera globally [1]. *Vibrio cholerae* (*V. cholerae*), serogroups O1 and O139 are the causative agents for cholera. Of the two serogroups, O1 is responsible for most epidemics in developing countries and is further classified into serotypes Ogawa, Inaba and Hikojima [2]. Ogawa and Inaba are the major serotypes, and these have been isolated in Zambia [3]. Transmission of cholera is mainly via consumption of contaminated water or food and in many endemic areas, children often suffer the highest disease burden [4].

In 2016, Zambia for the first time deployed the oral cholera vaccine (OCV) Shanchol™ (Shantha Biotechnics Private Limited, Hyderabad, India**)** [5]. The vaccine was WHO prequalified in 2011 [6]. While the vaccine has shown a protective efficacy of up to 66% in adults, very little is known about the factors that impact its immunogenicity in cholera endemic areas.

Oral vaccines are known for several distinct benefits compared with parenteral vaccines in that (i) they can be produced in large quantities at relatively low cost, (ii) are easy to administer and (iii) have the capacity to induce mucosal immunity in the gut, thereby directly protecting vaccinated individuals against subsequent infection. In addition, by blocking onward transmission, there is enhanced herd immunity [7, 8].

Notwithstanding all the potential benefits of oral vaccines, some genetic factors including histo-blood group antigens (HBGAs) have been implicated in the mechanistic cellular invasion of the host by pathogens. Some enteric pathogens and pathogen-derived toxins use the HBGAs as attachment factors [9]. For example, blood type O has been linked with an increased occurrence of paralytic poliomyelitis [10]. In the case of cholera, individuals with blood group O are less likely to be colonized by *V. cholerae* [11], but once colonized, are more likely to suffer severe symptoms [11, 12].

However, there isn’t sufficient evidence in literature on vaccine immunogenicity effects of these blood groups. Intriguingly, blood group O frequencies do not differ markedly in regions where oral cholera vaccines have underperformed compared to those in which a robust vaccine response is described [13, 14].

HBGAs may present on the surface of epithelial cells and in mucosal secretions such as saliva which is determined via fucosyl-transferase 2 (*FUT2*) gene. Individuals with functional *FUT2* gene facilitating secretion of HBGAs are referred to as secretors whereas those with mutations in *FUT2* that abrogates ability to secrete HBGAs as non-secretors respectively. In one report, non-secretors were more likely to suffer from severe cholera than secretors [15]. However, the extent to which the individual’s HBGA secretor status could influence vaccine immunogenicity remains unclear.

Given the high prevalence of blood type O among Zambians and the reports that individuals with blood group O are less likely to suffer from cholera [11], we hypothesized that there might be poor immune response to the new cholera vaccine, Shanchol™ among the Zambian population.

We thus set out to investigate the impact of HBGAs and secretor status among individuals residing in a cholera endemic area and assessed impact on immunogenicity to OCV.

## Methods

### Study design and participants

Using a longitudinal study, we used data from a clinical trial (ClinicalTrials.gov with trial # NCT04423159) that aimed at describing the longevity and waning of vibriocidal antibodies (IgG and IgM) after receiving two doses of OCV (Shanchol™) [16]. Thus, for the longitudinal study, we included all the participant data from this trial and dropped those with incomplete data at analysis.

The clinical trial was conducted in Lukanga Swamps of Central Province Zambia between October 2016 to October 2020. The trial enrolled 223 male and female adults aged 18 to 65 years, residing in Lukanga Swamps and who provided written informed consent to participate in the study. Individuals were excluded from participation if they had medical conditions such as hepatic disease, diarrhoea within the previous 7 days, had previously received OCV or were pregnant.

### Vaccination

All participants received two doses of Shanchol™ (Shantha Biotechnics Private Limited, Hyderabad, India), given 28 days apart. Participants were monitored for any adverse events following vaccination for 30 minutes after which they were allowed to go home. Upon release, participants were asked to return to the study site or to contact any study staff if they felt ill after receipt of the vaccine. All the vaccines were kept at 2-8 °C on site as recommended by the manufacturers.

### Sample collection

**Fig. 1:**
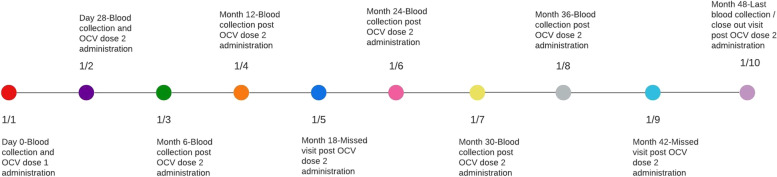
Schema for sample collection and vaccination time points

Participants who received both doses (at Day 0 and Day 28) were followed up for 48 months (Fig. 1). A 10 ml venous blood sample was collected after provision of written informed consent but prior to immunization on day 0. A second blood sample was collected before administering dose 2 at day 28. Thereafter, further blood samples were collected at months 6, 12, 24, 30, 36 and 48 accordingly. In addition, saliva was collected using SalivaBio collection kit (Salimetrics LLC) at month 12. All biological samples were processed and stored at − 80 °C pending sample processing. To avoid inter-assay variations, all samples for each individual were processed at the same time.

### Laboratory procedures

#### ABO blood typing

Was done by direct typing and reverse typing***.*** This test was carried out according to the manufacturer’s instruction (Biotec laboratories, UK). Briefly, one end of a slide was labelled Anti-A, the middle one labelled Anti-D and the other end Anti-B. A drop of Anti-A test serum was added to the end marked Anti-A, followed by Anti-D and a drop of Anti-B serum was added to the end marked Anti-B. One drop of whole blood was added to each end of the slide and mixed thoroughly using separate wooden toothpicks. The results were read directly from the slide within 60 seconds. A participant was reported to have blood type A if agglutination occurred with the Anti-A test serum; type B if agglutination occurred with the Anti-B test serum; type AB if agglutination occurred with both test serums, and O if there was no agglutination in either case. Blood group was also categorized as rhesus positive or negative if there was agglutination on Anti-D or not.

#### Secretor status testing

We determined salivary blood group ABO phenotypes and secretor status using an enzyme-linked immunosorbent assay (ELISA) based on published methods [17]. In brief, a 100 μL volume of saliva diluted to 1:100 in coating buffer (carbonate-bicarbonate buffer, p-H 9.6) was added to microtiter plate (Nunc-Immuno F96 Maxisorp, Thermo Fisher Scientific) and incubated at 37 °C for 2 hours followed by incubation at 4 °C overnight. Following overnight incubation, the plate was blocked with 3% weight/volume bovine serum albumin in phosphate buffered saline (PBS) and incubated at 37 °C for 1 hour. The plate was then incubated with 1:5000 diluted α-A (ABO1 clone 9113D10), and α-B (ABO2 clone 9621A8) (Diagast, France) monoclonal antibodies at 37 °C for 1.5 hours. Next, the plate was incubated with 1:7500 diluted peroxide-conjugated goat anti-mouse IgG (heavy and light chain) (Abcam, United Kingdom) for 1.5 hours at 37 °C. Color development was done using 3, 3′, 5, 5′ Tetramethyl benzidine (TMB) (Sigma Aldrich) substrate and stopped by addition of sulphuric acid before reading absorbance at 450 nm wavelength (BioTek). Throughout the assay, the microtiter plate wells were washed five times with 0.05% weight/volume Tween 20 in PBS (PBST) in between the ELISA steps. All antibodies were diluted in 5% fetal bovine serum (GE Healthcare Life Sciences, USA) in PBST. Secretor status was determined in a similar manner by detection of Fucα1-2Gal-R known to be present in secretors but not in non-secretors. Diluted saliva samples were coated to the microtiter plate and incubated overnight as described above. After blocking, the plate was incubated with peroxidase-conjugated *Ulex europaeus* (UEA-1) Lectin (Sigma Aldrich), developed with TMB substrate before addition of Sulphuric acid and absorbance read at 450 nm.

#### Vibriocidal antibody testing

Vibriocidal antibody assays was also run as previously described with some modifications [18]. Local Zambian *V. cholerae* O1 Inaba (EDVRU/ZM/091–10) and Ogawa (EDVRU/ZM/2016) were used. These strains were quality checked at Johns Hopkins University in the United States and their performance was comparable to standard strains Inaba (T19479) and Ogawa (X25049).

Briefly, colonies from overnight cultures were inoculated in Brain Heart Infusion (BHI) Broth and incubated at 37 °C for about 4 hours before harvesting the cells. Heat inactivated serum, exogenous guinea pig complement (Sigma Aldrich S1639-5ML) and *V. cholerae* bacterial cells were then placed in 96-well microtitre tissue culture plates (Life sciences, Durham, USA) and incubated at 37 °C. Vibriocidal titres were defined as the reciprocal of the highest serum dilution resulting in a 50% reduction in optical density read at 595 nm compared to positive control wells without serum. Seroconversion was defined as a 4-fold or greater increase from the baseline vibriocidal titres. A standard monoclonal antibody (mAb) and a high titre standard serum was used to normalize the results in case of inter-assay variations.

### Sample size calculation

The participants were selected from a convenience sample. All individuals satisfying inclusion criteria were considered for participation. Previously published data [19], suggested that at least, 80% adults would seroconvert. Due to marginal transience noted in this population it was estimated that 20% of those participating will be lost to follow-up. The sample size calculated with 95% confidence (α = 0.05 (two tailed)), 80% power (β = 0.2), a difference of 0.3 and conservative estimates of 0.5 variance for pre-vaccine and post-vaccine groups is 176. Adding 20% to account for anticipated attrition yields a total required sample size of 212 [20].

### Statistical analysis

Participants’ socio-demographic and clinical characteristics were presented as frequencies (percentages). Vibriocidal antibody titres were expressed as geometric mean titres (GMT), and sero-conversion rate was defined as the percentage of participants with at least a four-fold rise in serum vibriocidal antibody titres after vaccination on Day 28 HBGA phenotype was categorized into blood group O and non-blood group O. Equally, participants were categorised by secretor status as secretors or non-secretors. Seroconversion to Ogawa and Inaba were calculated for each background characteristic. Since the outcome was common (i.e., greater than 10% in the sample), we used Poisson regression with robust standard errors to determine the effect of HBGA phenotype and secretor status on vaccine uptake, with each adjusted for the other in addition to potential confounders such as socio-demographic, and WASH characteristics. Level of statistical significance was set at a 2-tailed *p*-value of 0.05 or less. Data analysis was performed using Stata 17 MP8 (StataCorp, College Station, TX, USA).

### Ethical approval

Ethical approval was obtained from the University of Zambia Biomedical Research Ethics Committee (UNZABREC) reference number 007–12-16, while the National Health Research Authority provided the authorization to conduct the study. Written informed consent was also obtained from all enrolled participants into the trial. All study procedures were conducted according to good clinical practice guidelines.

## Results

### Baseline characteristics of study participants

**Fig. 2:**
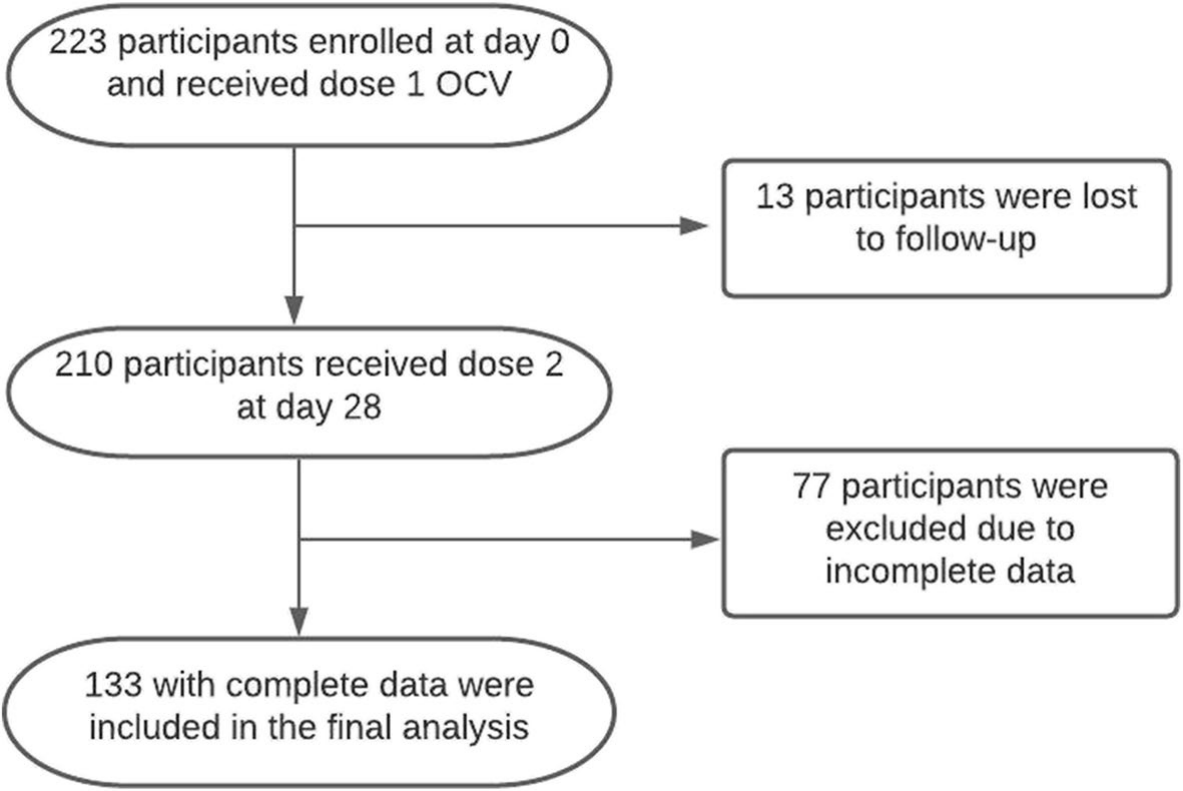
Flow diagram for study participant enrollments and follow-up

Of the 223 participants enrolled in the study, 13 were recorded as lost to follow-up as we could not track their way about and 77 were excluded due to incomplete data. A total of 133 were included in the final analysis (Fig. 2). Of the 133 participants, 76 (58%) were above 34 years old, 120 (90%) were male, 92 (72%) were engaged in fishing activities, 95 (81%) used water from the Swamps for drinking and 87 (74%) practiced open defecation on land (bush) or in the Swamps. Overall, there was no significant differences observed by sero-conversion rates to *V. cholerae* O1 Ogawa and Inaba using the baseline characteristics (Table 1).

**Table 1:** Sero-conversion rates by socio-demographic characteristics

Characteristics	Inaba			Ogawa		
	Total n(%)	n(%)Seroconverted	*p* value	Total n(%)	n(%) Seroconverted	*p* value
**Age (years)**^**a**^			0.805			0.728
16–24	27(20.5)	8(29.6)		27(20.3)	8(29.6)	
25–34	29(22.0)	8(27.6)		30(22.6)	8(26.7)	
> 34	76(57.6)	18(23.7)		76(57.1)	17(22.4)	
**Sex**						
Females	13(9.8)	1(7.7)	0.108	13(9.7)	2(15.4)	0.384
Males	120(90.2)	34(28.3)		121(90.3)	32(26.5)	
**Occupation**^**a**^						
Fishing	92(71.9)	23(25.0)	0.741	92(71.3)	24(26.1)	0.267
Fish Trading	16(12.5)	5(31.3)		17(13.2)	5(29.4)	
Others^b^	20(15.6)	4(20.0)		20(15.5)	2(10.0)	
**Source of drinking water**^**a**^						
Borehole/well	23(19.5)	6(20.0)	0.935	23(19.5)	3(13.0)	0.211
Swamps	95(80.5)	24(80.0)		95(80.5)	24(25.3)	
**Type of toilet facility**						
Swamp/bush	87(74.4)	21(24.1)	0.526	87(74.4)	21(24.1)	0.643
Pit latrine/Toilet	30(24.6)	9(25.8)		30(24.6)	6(20.0)	
**Total**	133(100)	35(26.3)		134(100)	34(25.4)	

^a^Total not equal to 133 or 134 because of missing information

^b^Chi square was used to compare sero-conversion by socio-demographic characteristics, blood group and secretory status

### Effects of blood group and secretory status on sero-conversion against Inaba

Of the 133 participants, 63 (47%) had blood type O based on direct typing, whereas 70 (53%) had a non-O blood type. Non-secretors were 14 (18%) while 62 (82%) were secretors (Table 2). The proportion of participants that seroconverted were higher among those with blood group O (30.2%) compared to non-O (22.9%) and among the secretors (35.7%) compared to non-secretors (25.8%). Participants with blood group O were about two times more likely to seroconvert against Inaba compared to the non-O blood groups, although this association was not statistically significant (Adjusted PR = 2.10, 95% CI: 0.79–5.60, *P* = 0.138). Furthermore, the non-secretors have a 41% higher chance of seroconverting against Inaba compared to the secretors; however, association was also not statistically significant (Adjusted PR = 1.41, 95% CI: 0.52–3.79, *P* = 0.496) (Table 2).

**Table 2:** Effects of blood group and secretor status on sero-conversion against Inaba

Characteristics	Total n (%)	n(%) Seroconverted	Inaba			
			Crude PR (95% CI)	***p*** value	Adjusted PR (95% CI)^**a**^	***p*** value
**Blood Group**						
Non-O	70 (52.6)	16 (22.9)	Ref		Ref	
O	63 (47.4)	19 (30.2)	1.32 (0.74–2.34)	0.343	2.10 (0.79–5.60)	0.138
**Secretor Status**^**b**^						
Secretor	62 (81.6)	16 (35.7)	Ref		Ref	
Non-Secretor	14 (18.4)	5 (25.8)	1.38 (0.61–3.16)	0.440	1.41 (0.52–3.79)	0.496

^a^Adjusted for age, sex, education, occupation, source of drinking water & type of toilet facility, PR- Prevalence Ratio

^b^Total not equal to 133 because of missing information

### Effects of blood group and secretory status on sero-conversion against Ogawa

The proportion of participants that sero-converted against Ogawa was the same among those with blood group O and the non-O blood group (25.4%) but higher among the secretors (35.7%) than the non-secretors (22.2%). The probability of seroconverting against Ogawa was 12% lower among blood group O participants compared to the non-O, although this association was not statistically significant (Adjusted PR = 0.78, 95% CI: 0.27–2.20, *P* = 0.633) while the non-secretors are about two times more likely to seroconvert against Ogawa compared to the secretors and this association was also not statistically significant (Adjusted PR = 1.91, 95% CI: 0.77–4.76, *P* = 0.166) (Table 3).

**Table 3:** Effects of blood group and secretor status on sero-conversion against Ogawa

Characteristics	Total n (%)	n(%) Seroconverted	Ogawa			
			Crude PR (95% CI)	***p*** value	Adjusted PR (95% CI)^**a**^	***p*** value
**Blood Group**						
Non-O	71 (53.0)	18 (25.4)	Ref		Ref	
O	63 (47.0)	16 (25.4)	1.00 (0.56–1.80)	0.995	0.78 (0.27–2.20)	0.633
**Secretor status**^**b**^						
Secretor	63 (81.8)	14 (35.7)	Ref		Ref	
Non-secretor	14 (18.2)	5 (22.2)	0.48 (−0.55–1.50)	0.362	1.91 (0.77–4.76)	0.166

^a^Adjusted for age, sex, education, occupation, source of drinking water & type of toilet facility, PR- Prevalence Ratio

^b^Total not equal to 134 because of missing information

### Kinetics of vibriocidal GMTs against *V. cholerae* O1 Inaba and O1 Ogawa overtime by blood group

**Fig. 3:**
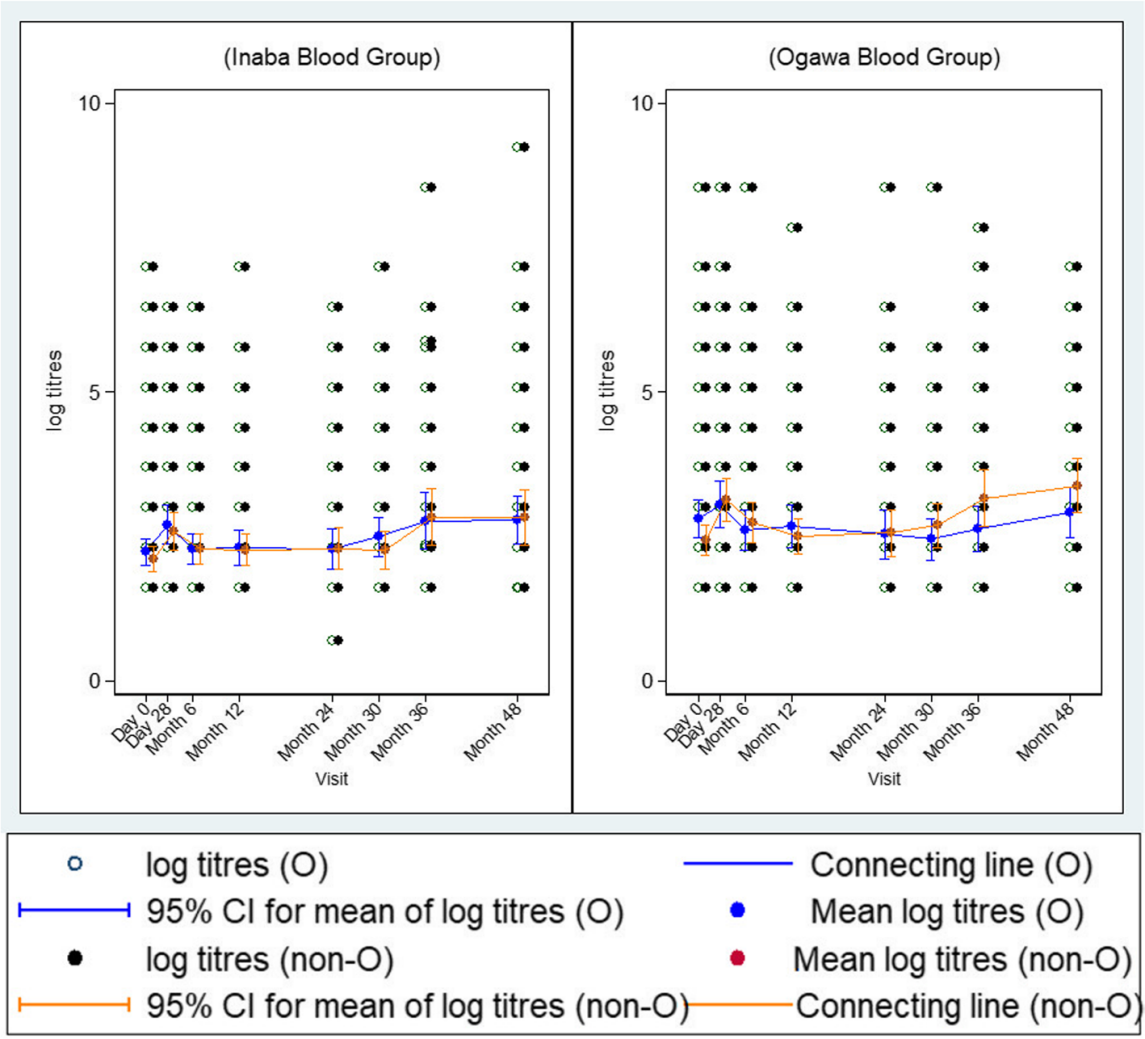
Kinetics of vibriocidal GMTs at different time points (left) *V. cholerae* O1 Inaba (right) *V. cholerae* Ogawa for blood type O and non-O. There was a rise in antibody titres in both serotypes at day 28 and then started to decline close to baseline titres at month 12. A significant but comparable rise was observed at months 36 and 48

Overall, we observed insignificant increases in vibriocidal GMTs (95% CI) by both non-O and O blood group types at baseline, [2.44 (2.18–2.70)] and [2.81 (2.48–3.14)] and day28, [3.12 (2.75–3.49)] and [3.05 (2.65–3.46)] against *V. cholerae* O1 Ogawa as shown in Fig. 3. Thereafter, titres dropped steadily up to month 24 after which a gradual increase was again observed from month 30 up to month 48. Although non-O individuals showed a higher trend in terms of recorded titres than blood group O starting at month 30, the differences in titres were not statistically significant between the two groups. The trend in vibriocidal GMT (95% CI) against *V. cholerae* O1 Inaba between the two groups (non-O and O blood types) were similar with that observed for O1 Ogawa (Fig. 3).

### Kinetics of vibriocidal GMTs to *V. cholerae* O1 Inaba and O1 Ogawa overtime by secretor status

**Fig. 4:**
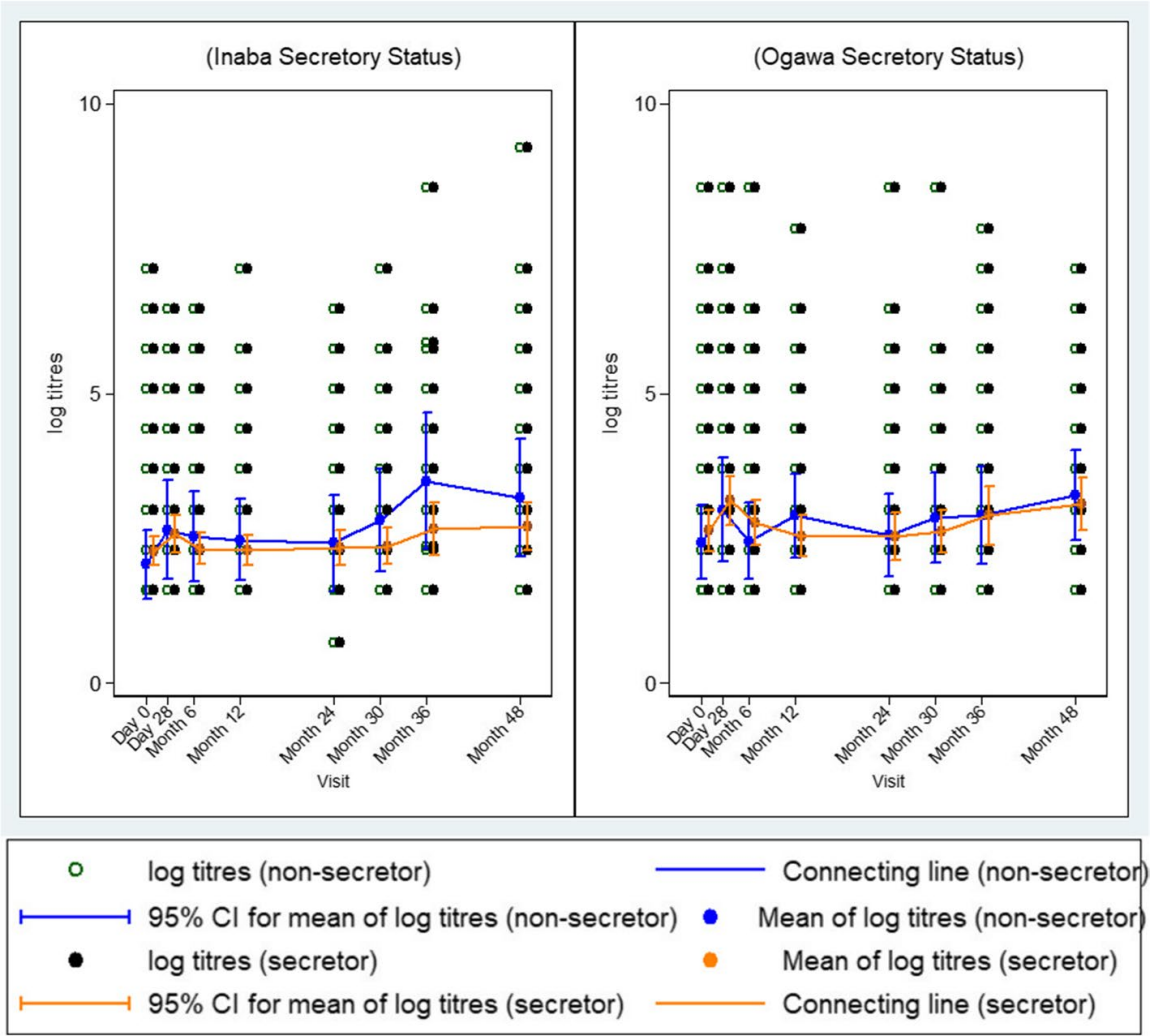
Kinetics of vibriocidal GMTs at different time points (left) *V. cholerae* O1 Inaba (right) *V. cholerae* Ogawa for secretors and non-secretors. There was a rise in antibody titres in both serotypes at day 28 and then started to decline close to baseline titres at month 12. A significant but comparable rise was observed at months 36 and 48

Furthermore, we also noted that baseline GMTs (95% CI) were lower in both the non-secretors 2.43 (1.80–3.06) and the secretors 2.63 (2.27–2.99) but increased slightly at Day 28 in both the non-secretors 3.00 (2.11–3.88) as well as the secretors 3.15 (2.73–3.57) against *V. cholerae* O1 Ogawa as shown in Fig. 4. Similar to blood type trends, after Day 28, GMTs started to drop until month 24. Beyond 24 months, there was a constant increase observed from month 30 up to month 48. Although non-secretor individuals showed a higher trend in terms of recorded titres than the secretors, the differences in titres were not statistically significant between the two groups. The trend in vibriocidal GMT (95% CI) against *V. cholerae* O1 Inaba between the two groups (non-secretors and secretors) were similar with that observed for O1 Ogawa (Fig. 4).

## Discussion

Our study found that ABO blood groups as well as secretor status do not impact OCV immunogenicity and seroconversion in the studied population.

The GMT kinetics were similar in either group i.e. non-O and O as well as secretor and non-secretor. We also found that in our study population, majority of our participants were secretors similar to what is reported by Arifuzzaman and colleagues [21]. Specifically, GMTs in all the vaccinees, either belonging to blood type O or non-O were comparable. This finding is similar to what was reported by Ramamurthy and colleagues [22]. Despite the supposed frequency that people with non-O may suffer from cholera [11], and assuming that each exposure boosts immunity against cholera, our results demonstrate that both individuals with O blood type and the non-O blood type elicit similar vibriocidal antibody responses to Shanchol™ in an area where cholera is endemic. While our study was underpowered to show a statistical difference, the data shows no definite trend to distinguish O from non-O blood types in terms of vaccine immunogenicity; as such vaccination campaigns should target all individuals regardless of their blood type. This is contrary to what was found elsewhere [23], as our vibriocidal antibody response did not differ between O and non-O individuals. The referenced trial conducted in Bangladeshi on the role of ABO blood group and efficacy of an oral, killed cholera vaccine, found considerably lower protection in recipients who were blood group O [23]. The implication here is that inactivated oral vaccines may not necessarily provide less protection to individuals with O blood-type. Further to this, another study conducted in Bangladeshi among children allocated to receiving a live, oral attenuated cholera vaccine, found higher frequency of serological responders in A blood group compared to the O blood group in the studied children [24].

The study by Arifuzzaman and colleagues reported that non-secretor individuals were more susceptible to *V. cholerae* O1 disease compared to the secretors [21]. However, in our population, we could not report on susceptibility to *V. cholerae* O1 disease as we did not collect stool samples to screen for asymptomatic infections.

Nonetheless, we postulated that if there is similar response to the vaccine in the non-secretors and the secretors, it is unlikely that in our population non-secretors would be more susceptible to cholera. Further, our study showed that non-secretors generally had higher GMTs against O1 Inaba when compared to the secretors. It is thus plausible that non-secretors were more susceptible to *V. cholerae* O1 Inaba serotype and could be why they generated higher titers. Consequentially, this result could then be seen as consistent with Arifuzzaman *et al* who reported non-secretors having an increased risk of symptomatic cholera [21]. However, we add caution to such interpretation as several factors can influence susceptibility to *V. cholerae* including, but not limited to lack of immunity on encountering the organism [11, 25], nutritional deficiencies [26, 27] and human genetic polymorphisms [28].

Seeing that both the O1 Ogawa and the O1 Inaba cholera serotypes circulate in Zambia [3], our finding of higher GMTs against O1 Inaba serotype could also mean that during the period of study, O1 Inaba serotype was the most circulating strain that people might have been exposed to. However, reports by Mwape and colleagues indicate that in 2016, the most circulating cholera serotype isolated during the cholera outbreak was O1 Ogawa [3].

Vibriocidal antibody titers have been reported to be a useful marker for measuring immunologic response including seroconversion to cholera vaccines [29, 30]. With this assay we found comparable seroconversion rates in our study population either by blood type or secretor status. Seroconversion rates also did not differ by *V. cholerae* serotypes. Nonetheless, Charles et al. found that individuals with blood type O had greater vibriocidal GMTs against serotype Inaba compared to those with non-O blood types [31]. Similar to our study, there was no significant difference observed for vibriocidal response to the Ogawa serotype in the same population [31]. Timing of the blood sampling in our study was further away from the vaccination date compared to Charles and colleagues that collected and measured GMTs 7 days after every dose [31]; this may be important given that vibriocidal antibody titers are known to wane quickly following vaccination as demonstrated by other researchers [32, 33].

We further observed the trends in GMTs over time and found no differences either by blood type and secretor status. We found that vibriocidal GMTs increased by Day 28 and begun waning beyond that. Although similar trends in vibriocidal GMTs have been reported by Harris et al. these only go as far as 1 year [33], while we are reporting here kinetics through 48 months of follow up.

Additionally, Harris and colleagues report that by 1 year, vibriocidal antibodies return to baseline levels [33]. In our study, we observed a reduction in titer getting to the lowest about 20 months post vaccination, but they remained slightly higher than baseline levels, and then began to rise beyond 30 months remaining above Day 28 levels at month 48. Part explanation for observed differences could be that there was ongoing exposure in our study population, and hence the raise we see beyond month 30 is actually a boost of vaccine response by subclinical natural exposure. A compelling environmental consideration from our study population is that they live on a swamp where open defecation is a norm, and with limited clean water sources, residual exposure is very likely. While difficult to be conclusive, it is also plausible that vaccination campaign could have conferred herd immunity and therefore the community remains relatively protected from a typical outbreak.

Finally, we also noted that generally dose two was not as beneficial in that there was a reduction in immune responses as seen in another similar study [34].

A major strength of this study is that it’s the first to measure genetic factors in individuals vaccinated against oral cholera vaccine Shanchol™ in our region, and the longest follow up we have seen in literature.

The major weaknesses for this study are first, the small sample size. The computed sample size was 212, but the study ended up with only 133 included in this analysis and this could explain the wide confidence margins and potentially why the observed differences were not statistically significant. Secondly, we did not collect stools to also assess for helminth infections which have been found to impair the immune response to oral cholera vaccine, particularly in non-O blood groups [35]. Thirdly, we imagine that our results may have been skewed because our target was vulnerable men and women involved in fishing activities and it turned out that most of our participants were men. Fourth, because this was a nested study and used data for the clinical trial, we did not have our own eligibility criteria but relied on what the parent study applied on enrolled participants.

## Conclusion

Several factors that influence susceptibility to cholera may affect responses to cholera vaccines. Our study has failed to conclude that the ABO system or the secretor status do have an impact on vaccine response. Future studies to address this question would be required to settle the questions comprehensively and allow for result generalizability.

In the absence of conclusive data, individuals in endemic areas at risk of cholera need to be vaccinated equally, regardless of the blood type or secretor status. Longevity of protection by the vaccine requires specific research in order to inform policy on when to revaccinate in previously immunized communities which remain at risk of outbreaks.

## Data Availability

Data will be made available to any interested researchers upon request. The CIDRZ Ethics and Compliance Committee is responsible for approving such request. To request data access, one must write to the Secretary to the Committee/Head of Research Operations, Ms. Hope Chinganya (Hope.Chinganya@cidrz.org). Dataset request must include contact information, a research project title, and a description of the analysis being proposed as well as the format it is expected. The requested data should only be used for the purposes related to the original research or study. The CIDRZ Ethics and Compliance Committee will normally review all data requests within 48–72 hours (Monday - Friday), and provide notification if access has been granted or additional project information is needed, before access can be granted.
